# Sertraline Induces Toxicity and Behavioral Alterations in Planarians

**DOI:** 10.1155/2017/5792621

**Published:** 2017-05-24

**Authors:** Isabela Salvador Thumé, Marcos Emílio Frizzo

**Affiliations:** Laboratory of Cellular Neurobiology, Department of Morphological Sciences, Universidade Federal do Rio Grande do Sul, Porto Alegre, RS, Brazil

## Abstract

Toxicity attributed to sertraline has been demonstrated recently in different cell types and also in some organisms. We investigated the effect of sertraline on planarians, which are considered suitable for investigations in neurotoxicology and currently are widely used as an animal model in neuropharmacological studies. Planarians treated with 10 *µ*M sertraline showed a rapid reduction in their spontaneous movement until they became completely motionless and then showed a series of asynchronous paroxysms (seizures) followed by progressive tissue damage, beginning 48 h after the sertraline treatment, and died approximately 72 h later. Our data showed that sertraline does not cause planarian death within the range of therapeutic concentrations; however, behavioral alterations were observed with concentrations that can be considered compatible with therapeutic ones, such as a significant reduction in planarian locomotory activity at 0.4 *µ*M. Treatment with 4 *µ*M sertraline had a significant effect, reducing planarian locomotory activity and increasing the number of asynchronous paroxysms; both effects were significantly maintained even 24 h after the sertraline was withdrawn. These behavioral changes observed at low micromolar concentrations suggest that sertraline might have residual biological consequences for planarians, even after it is withdrawn.

## 1. Introduction

Sertraline (Zoloft) is a selective serotonin reuptake inhibitor that is widely prescribed as an antidepressant [1] and is also used for the treatment of panic, obsessive-compulsive, and posttraumatic stress disorders [2]. Beyond its classic mechanism of action, extensive evidence suggests that sertraline possesses other biological activities that appear to be unrelated to its inhibition of serotonin reuptake [3]. Among these additional biological effects, sertraline was recently reported to inhibit Na^+^ and K^+^ voltage-dependent channels [3–9] as well as to reduce glutamate uptake [10]. Regarding an effect of sertraline on Ca^2+^ channels, it was recently reported that it can induce a rise in the levels of cytosolic free Ca^2+^ partly through these channels and also through endoplasmic reticulum release in cancer cells [11, 12], where it also induced apoptosis [12, 13]. Although sertraline is considered a safe drug, several reports have associated it with toxic effects on the liver [14–16] and hepatic cells [2, 13, 17]. Recently, cardiac toxicity has been related to sertraline, even in therapeutic doses [18, 19], and its use during pregnancy was associated with an increased risk of major malformations [20] and compromise of embryonic bone development [21].

Recently, planarians have been proposed as an alternative model for investigations in neurotoxicology [22, 23], showing sensitivity to chemicals comparable to other animal models used in toxicological studies, such as zebrafish larvae and nematodes [22]. Planarians possess an aggregate of neurons in the head that are very similar to those of vertebrates. The major shared similarities include the multipolar shape, dendritic spines with synaptic boutons, a single axon, and relatively low spontaneously generated electrical activity ([24]; for review see [25]). Planarians also possess several neurotransmitter-receptor systems and are widely used as an animal model in neuropharmacological studies [25]. For instance, they present a serotonergic system that share some similarities with those found in vertebrates [25] and where serotonergic drugs showed effect on both spontaneous locomotor velocity and behavior [26].

In view of the recent reports of sertraline toxicity, we decided to investigate this issue employing a well-established animal model that is used in pharmacological and toxicological studies. We investigated if sertraline could affect planarian behavior and cause toxicity in these animals.

## 2. Methods

### 2.1. Drug and Materials

Sertraline (Sigma-Aldrich, St. Louis, Missouri, USA) was dissolved in sterile water and stored as stock solutions. On the day of the experiment, stock solutions were diluted to desired concentrations in artificial pond water (APW) (deionized water, 6 mM NaCl, 0.1 mM NaHCO_3_, and 0.6 mM CaCl_2_). An HDL HM-Pro 480 video camera (Amazonas, Brazil) with a manual iris varifocal lens (2.8–12.0 mm, sensitivity 0.5 lux), Greatek GTK-DVR04A, H.264, NTSC digital video recorder (São Paulo, Brazil), square open-top plastic cube container (6 cm × 6 cm), HT-2 hand tally counter (Taiwan, ROC), and electronic timer (VWR International, Radnor, PA, USA) were used in these studies. Focal length was maintained at 10 cm.

### 2.2. Subjects

Planarians* (Dugesia tigrina)* obtained from the Laboratory of Platyhelminthes (Department of Zoology, UFRGS, Brazil) were stored in Tupperware containers at 21°C in the dark. Animals were fed organic beef liver once a week, and the water (APW) in their container was changed twice a week.

### 2.3. Procedures

The protocol used is standard for behavioral experiments and had a duration of 72 h. During the experimental period, each planarian was maintained at room temperature (21°C) under a 12 h dark-light cycle, in a clear plastic square dish (6 cm × 6 cm) filled with 9 mL APW. Planarians 1.0–1.5 cm in length, chosen randomly, were fed 24 h before the experiment and were not fed at any time during the experimental procedures. Each planarian was used only once and remained in the test dish until the conclusion of the experiment. When necessary, the medium (APW or APW containing the drug) was replaced with a syringe. The video record was made from the top of the cube. Each planarian was studied individually during 72 h, with the following standard protocol: (1) transference to the test dish; (2) 10 min for acclimation; (3) 10 min of recording (considered as control); (4) replacement of medium with sertraline; (5) 25 min of incubation; (6) 10 min of recording (considered as sertraline 25 min); (7) 24 h of incubation; (8) 10 min of recording (considered as sertraline 24 h); (9) replacement of incubation medium with APW (5x); (10) 24 h of incubation in APW; (11) 10 min of recording (considered as sertraline withdrawal, 24 h). Hence, the test groups were the following: control, 25 min sertraline and 24 h sertraline, and withdrawal, 24 h. All planarians were used in each group. In order to determine the possibility of environmental and/or physical interference with the planarians during the protocol execution, a pilot evaluation was carried out before the sertraline was introduced. In this group, planarians were exposed to the same conditions described above (72 h at 21°C, 12 h dark-light cycle, and the same exchanges of medium) but were maintained in APW medium throughout.

### 2.4. Behavioral Studies

#### 2.4.1. Determination of Locomotory Activity

In APW, planarians exhibit normal gliding behavior. To quantify spontaneous planarian movement (pLMV), individual planarians were placed in a square dish that was then placed on paper with gridlines spaced 0.5 cm apart. pLMV was quantified as the number of gridlines crossed or recrossed during a 10 min observation period. Normal planarian movement consists of constant-velocity, horizontal, and forward-directed movement, with periodic turns, but rarely any stops [27].

#### 2.4.2. Determination of Seizure Events

Planarian seizure-like activity (pSLA) is defined as asynchronous paroxysms resulting in a sudden disruption of normal spontaneous locomotor activity, in response to proconvulsive drugs [28, 29]. The behaviors associated with pSLA are described as headswing (planarian stationary, tail portion fixed to ground with head portion moving about from side to side); C-like position (CLP) (planarian adopts a C-like shape); and screw-like hyperkinesia (SLH) (planarian adopts a screw-like shape with a twisting about the longitudinal axis) [30–32]. In this study, each video recording made to quantify pLMV was also analyzed to identify pSLA. The asynchronous C-like, SLH, and headswing paroxysms were quantified together as total pSLA during 10 min (the number of events counted). In this case, overt behaviors of planarians were also photographed to illustrate the behavioral response. The records were analyzed by two observers, one of them blinded to the group observed.

### 2.5. Data Analysis and Statistical Methods

Data were expressed as mean ± SEM (*n* = 5). Cumulative pLMV for each test group was expressed as a percentage of the mean pLMV of the control group (step (3) described in Procedures in Section 2.3). Comparisons of group means were determined by two-way ANOVA followed by Tukey's multiple comparisons test (GraphPad Prism, 6.01). Statistical significance was assumed at *p* < 0.05.

## 3. Results

**Figure 1:**
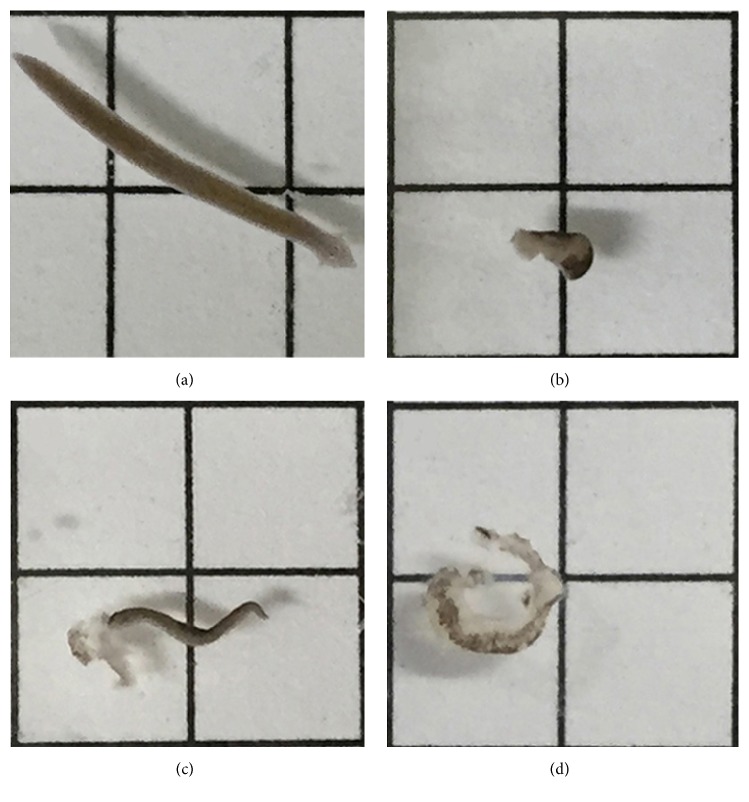
Toxicity mediated by sertraline in planarians. In (a), a representative image of a control planarian during its gliding. (b) shows a planarian after 1 h of exposure to 10 *µ*M sertraline, and (c) and (d) show planarians after 72 h of exposure.

Untreated planarians exhibited normal gliding behavior (Figure 1(a)), which was quantified as spontaneous planarian movement (pLMV). In order to investigate a potential toxicity of sertraline, planarians were exposed to different concentrations (0.1 to 10 *µ*M) for two weeks. During this period, no evidence of a deleterious effect was observed with the lower sertraline concentrations (0.1 to 4 *µ*M), despite overt alterations in the behavior of planarians maintained at 1 and 4 *µ*M. However, when treated with the highest concentration (10 *µ*M), the pLMV was drastically reduced around 15 min later, and the planarians underwent an intense and continuous series of seizures around 45 min after exposure. After 60 min of exposure to 10 *µ*M sertraline, the animals showed an absence of pLMV (Figure 1(b)) and repeated episodes of seizures. Continuous exposure for 72 h evoked partial (Figure 1(c)) or widespread damage (Figure 1(d)). A deleterious effect of 10 *µ*M after an exposure time of 72 h was detected in all planarians tested, although the effect was more pronounced in some planarians than in others. The tissue injury was progressive and resulted in debris sloughed from the degenerating planarian body.

**Figure 2:**
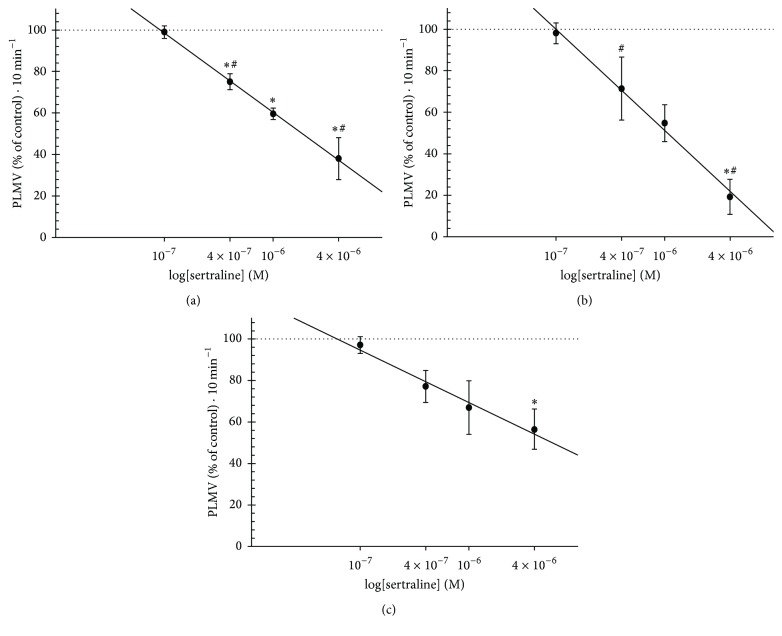
The effect of sertraline on planarian locomotor activity. The effect of different sertraline concentrations on the spontaneous planarian movement (pLMV) was quantified following sequential incubations of 25 min (a), 24 h (b), and 24 h after sertraline withdrawal (c). Dotted line represents control levels. Data are expressed as mean ± SEM from 5 independent experiments (*n* = 5). *∗* indicates being significantly different from the control; # indicates being significantly different from each other (*p* < 0.05).

The effect of sertraline on the animals' locomotion was studied through quantification of spontaneous planarian movement (pLMV). The effect of different sertraline concentrations (0.1 to 4 *µ*M) on pLMV was studied acutely (25 min), after 24 h of exposure, and then 24 h after the sertraline was withdrawn. As depicted in Figure 2(a), when planarians were exposed to sertraline for 25 min, pLMV were significantly reduced compared to the control, to 75.1 ± 3.8% (0.4 *µ*M), 59.6 ± 2.7% (1 *µ*M), and 38.0 ± 10.1% (4 *µ*M), *p* < 0.05, *r*^2^ = 0.999. Exposure to sertraline for 24 h significantly reduced pLMV in respect to control to 54.7 ± 8.9% (1 *µ*M) and 19.1 ± 8.4% (4 *µ*M), *p* < 0.05, *r*^2^ = 0.993 (Figure 2(b)). The withdrawal of sertraline after 24 h of treatment significantly reduced pLMV in respect to control to 56.5 ± 9.7% (4 *µ*M) even if 24 h after the drug has been withdrawn, *p* < 0.05, *r*^2^ = 0.974 (Figure 2(c)).

**Figure 3:**
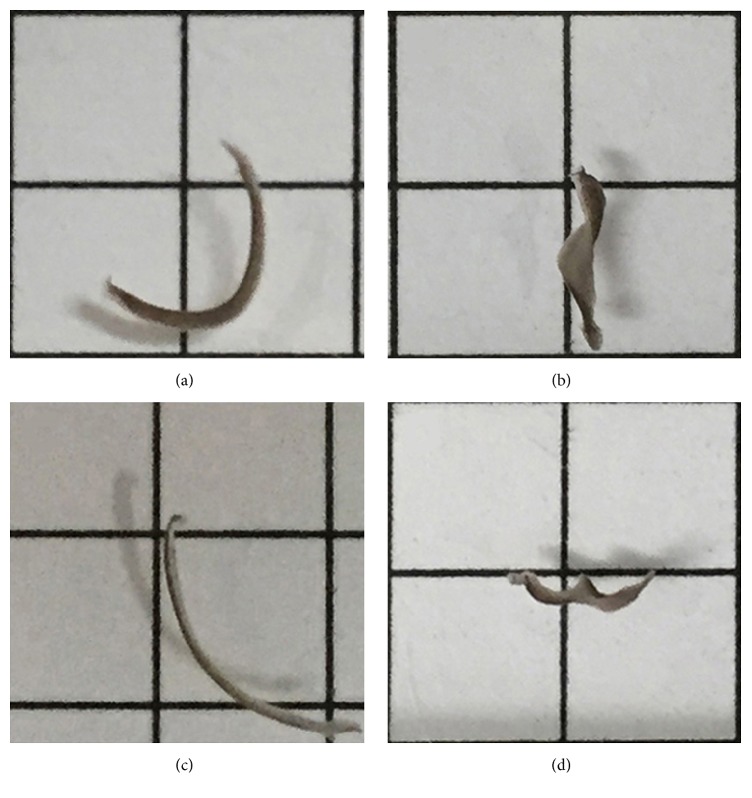
Planarian seizure-like activity evoked by sertraline. The planarian images were made after 24 h of exposure to 1 *µ*M sertraline ((a) and (b)) and 4 *µ*M sertraline ((c) and (d)). The asynchronous paroxysms C-like position ((a) and (c)) and screw-like hyperkinesia ((b) and (d)) were observed for both sertraline concentrations.

Sertraline exposure reduced pLMV and also elicited overt alterations in the planarian shape that were recognized as asynchronous paroxysms and were photographed for records (Figure 3). These changes in shape were not observed in untreated planarians submitted to the same protocol, as previously mentioned in Procedures in Section 2.3, or before the treatment with sertraline.

**Figure 4:**
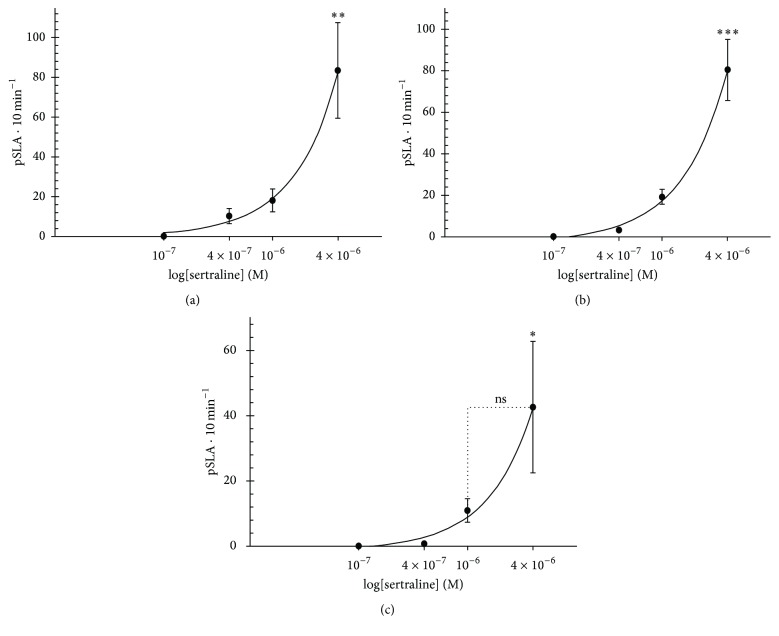
Quantification of planarian seizure-like activity evoked by sertraline. The effect of different sertraline concentrations on the seizure-like activity (pSLA) was quantified following sequential incubations of 25 min (a), 24 h (b), and 24 h after sertraline withdrawal (c). Data are expressed as mean ± SEM from 5 independent experiments (*n* = 5). The asterisks mean being significantly different from the other groups: ^*∗*^*p* < 0.05, ^*∗∗*^*p* < 0.01, and ^*∗∗∗*^*p* < 0.001 (ns means not significant).

The asynchronous paroxysms observed during 10 min of the recording period were counted as total planarian seizure-like activity (pSLA) (Figure 4). The lowest sertraline concentrations used (0.1–1 *µ*M) were not statistically different from the control. After 25 min of incubation with 4 *µ*M sertraline, the planarians showed a significant increase in the number of pSLA (Figure 4(a)), which remained significantly higher after 24 h of incubation with sertraline (Figure 4(b)). Notably, sertraline (4 *µ*M) withdrawal did not abolish the significant increase of pSLA even after 24 h (Figure 4(c)). The effect of 4 *µ*M sertraline in increasing pSLA was significant for the lower concentrations in all incubation times (25 min, 24 h) and also 24 h after withdrawal (except for 1 *µ*M). Additionally, the rise in the pSLA elicited by this concentration was similar in all three experimental conditions (Figures 4(a), 4(b), and 4(c)).

## 4. Discussion

The cytotoxicity of sertraline has been described in different cell types for concentrations within a *μ*M range, although the degree of sensitivity of these cell types seems to differ. For instance, considering a sertraline incubation of 24 h, cytotoxicity was observed from 20 *µ*M in human prostate-cancer cells (PC3) [12] as well as in osteosarcoma cells (MG63) (Lin et al., 2013), from 25 *µ*M in a human hepatoma cell line (HepG2) [13], from 30 *µ*M in human platelets [10], and from 37.5 *µ*M in rat primary hepatocytes [2]. Additional evidence regarding sertraline toxicity has been reported in some organisms, such as the parasite* Leishmania donovani* [33], fish, and aquatic invertebrates [34]. In our study, sertraline (10 *µ*M) proved to be toxic to planarians when the exposure time exceeded 48 h. In this condition, progressively deleterious consequences were observed, resulting in death after around 72 h. Debris was sloughed from the animal's body during the degenerative process.

Our data showed that sertraline does not kill planarians at therapeutic concentrations, which are considered to be <1 *µ*M [35]. For instance, Saletu et al. [36] reported that a single oral dose of 400 mg sertraline administered to healthy volunteers resulted in a plasma concentration of ~0.74 *µ*M, while another study carried out with adults treated with 25–150 mg once a day for a month found that the plasma sertraline level was 8 nM–0.32 *µ*M [37]. However, in our study, behavioral alterations were observed with sertraline concentrations that can be considered compatible with the therapeutic ones. Treatment with sertraline evoked a significant and concentration-dependent reduction in the planarian locomotory activity from 0.4 *µ*M. This inhibitory effect was significantly higher with 4 *µ*M and was also significantly different from the control even 24 h after the sertraline was withdrawn. This sertraline concentration also evoked a significant increase in the number of asynchronous paroxysms, with respect to the control and the other concentrations used. The stimulatory effect on planarian seizure-like activity remained significantly increased even 24 h after the sertraline was withdrawn. These behavioral results are interesting since they correspond to overt and complex effects that were observed at low micromolar sertraline concentrations. They also suggest that sertraline might have biological consequences that persist even after it is withdrawn, which might be explained by its capacity to accumulate in the brain tissue [38, 39].

In rodents, serotonin receptors are related to a variety of stereotyped behaviors such as “head shakes,” “backpedaling,” “head-twitch,” “head-weaving,” “turning,” and “forepaw treading” [40–42]. Considering that previous works in planarians showed an effect of serotonergic drugs on both spontaneous locomotor velocity and behavior [26], one could suppose that sertraline effect could be related to the behavioral alterations here described. In this case, bearing in mind the sertraline's classic mechanism of action, the inhibition of serotonin reuptake might increase the neurotransmitter level and consequently mediate an endogenous activation of their receptors. However, it is important to consider that, in these studies where behavioral effects of serotonin-addition were observed, the concentration of the neurotransmitter added was high, around 100–1000 *µ*M [26, 43]. Another additional possibility to be considered might be an indirect effect of sertraline on other neurotransmission systems, such as the glutamatergic system.

The biological effects of sertraline seem to be more complex than its traditionally described mechanism of action. It has been recently shown that it may cause inhibition of different types of Na^+^ and K^+^ channels [3–8]. An inhibition of Ca^2+^ channels mediated by sertraline has also been reported in excitable cells [6], but its effect on these channels seems to be different in other cell types [12]. Several studies [9, 11, 12] found that it can induce an influx of Ca^2+^ via store-operated Ca^2+^ channels in nonexcitable cells. An additional effect of sertraline was recently described in human platelets, where it caused a reduction in glutamate uptake [10].

Recently, several authors have attributed a wide range of biological effects to sertraline, suggesting that many of these effects are unrelated to its action as an inhibitor of serotonin reuptake [3, 5, 7, 9, 11, 12]. Ohno et al. [7] suggested that these additional biological actions may be implicated in the therapeutic and/or adverse effects of sertraline. Ohno et al., [7] also showed that sertraline inhibits K^+^ channels, such as the astroglial inwardly rectifying channel Kir4.1, which is responsible for astroglial K^+^ buffering; and Kobayashi et al. [5] found that it inhibits the G protein-activated inwardly rectifying K^+^ channels, Kir3. Our results demonstrated that, in low concentrations, sertraline evokes a planarian seizure-like activity a few minutes after exposure. We hypothesize that this effect may be due to a potential inhibition of K^+^ channels, such as Kir4.1. The blockade of the astroglial Kir4.1 channels was related to reduction of both K^+^ buffering and glutamate uptake, which could potentially lead to hyperexcitability of neurons and seizure activity [7, 44–46].

In summary, to our knowledge this is the first study to investigate the effect of sertraline on behavior and also its toxicity in planarians. The results demonstrate that sertraline, over a low micromolar range, affects spontaneous locomotory velocity, evokes episodes of asynchronous paroxysms, and may be toxic to these animals. Notwithstanding, additional experiments are still necessary to test our hypothesis that the effects observed on planarians were due to an action of sertraline on K^+^ channels.

## Supplementary Material

Video showing planarian behavior in four different conditions: (a) before sertraline; (b) after 25 min of incubation with 4 µM sertraline; (c) after 24 h of incubation with 4 µM sertraline; and (d) 24 h after sertraline withdrawal. For each condition, 1 min of the original 10-min recording is shown.
